# Editorial: Comorbidities in Psoriatic Arthritis and Their Impact on Therapeutic Strategies

**DOI:** 10.3389/fmed.2021.830179

**Published:** 2022-02-03

**Authors:** Ilenia Pantano, Piero Ruscitti

**Affiliations:** ^1^Rheumatology Unit, Department of Precision Medicine, University of Campania L. Vanvitelli, Caserta, Italy; ^2^Department of Biotechnological and Applied Clinical Sciences, University of L'Aquila, L'Aquila, Italy

**Keywords:** psoriatic arthritis, comorbidities, BDMARDs, cardiovascular disease, obesity

Psoriatic Arthritis (PsA) is a chronic disease characterized by the inflammatory involvement of joint in patients with psoriasis, manifest or not (1). PsA may be also associated with a significant rate of comorbidities, mainly cardiometabolic, which, together with the musculoskeletal manifestations, may have a relevant impact on quality of life and outcome of these patients (2, 3). The synergy between inflammation and “traditional” cardiovascular risk factors may result in this typical clinical phenotype and may be considered as a part of the psoriatic disease itself. Multiple lines of evidence may suggest that at the basis of these comorbidities there is a cytokine activation (2, 3). The systemic inflammation is pivotal in the pathogenesis of atherosclerosis, involving low-grade inflammatory activity in the vascular wall (4). Moreover, inflammatory cytokines, such as TNF and IL-6, are also involved in the pathophysiology of hypertension and dyslipidemia associated with obesity and insulin resistance. In recent years, several drugs have been approved for PsA but little data is available on their impact of on associated comorbidities of these patients. Similarly, the impact of these comorbidities in influencing the choice as well as the efficacy of such medications remain to be fully evaluated. There are no specific data which may guide the physician in the therapeutic choice based on the presence of certain comorbidities. Given that the comorbidities may be linked to the inflammatory process underlying PsA, it could be possible to speculate that the currently available therapies could also play a role in the associated disorders.

Taking together these observations, in this special issue, the impact of comorbidities was assessed in the management of patients with PsA. The selected articles are very different from each since the topic itself is very heterogeneous. The association between PsA and different comorbidities was discussed, highlighting the relevance of this problem in managing these patients (Lu et al.; De Lorenzis et al.; Alonso et al.; Atzeni et al.; Novelli et al.; Chia et al.; Tripolino et al.; Ramírez et al.; Englbrecht et al.). The rate of depression was discussed in PsA, which is acknowledged as a frequent comorbidity in inflammatory arthritis influencing the patient clinical picture (Englbrecht et al.). In addition, a vascular dysfunction was observed in patients with PsA and depression; a correlation between flow mediated dilatation features and depressive symptoms was observed (De Lorenzis et al.). The most frequently topic of these works was the cardiometabolic risk associated with PsA (Atzeni et al.; Novelli et al.; Ramírez et al.). An update about cardiovascular risk in PsA was provided, also describing the involved inflammatory pathogenic mechanisms (Novelli et al.). In fact, a number of epidemiologic studies, systematic reviews, and meta-analyses have suggested that PsA may be considered an independent risk factor for major adverse cardiovascular events. In addition, the clinical impact of obesity was reviewed in PsA (Ramírez et al.); this is recognized as an independent risk factor for PsA occurrence and its association with cardiometabolic burden (2). The clinical role of hyperuricemia in managing patients with PsA was also pointed out. Serum levels of uric acid may be associated with the severity of clinical manifestations and inflammatory features in patients with PsA (Tripolino et al.). In addition, in this special issue, the treatment was described in PsA with associated extra-articular manifestations or comorbidities (Alonso et al.; Atzeni et al.; Tripolino et al.). The management of concomitant uveitis and inflammatory bowel disease in these patients may be associated with specific therapeutic strategies according to different underlying pathogenetic steps (Chia et al.). Frequently, patients with PsA are characterized by the presence of metabolic syndrome, but the impact of approved therapies on that is far from being optimal. Thus, the main evidence related to the possible effects of synthetic and biologic DMARDs on metabolic syndrome outcomes was described in patients with PsA (Atzeni et al.). Finally, the effectiveness of secukinumab, a monoclonal antibody binding IL-17A, was explored in a “real-life” setting (Alonso et al.). Interestingly, in this study, the presence of some comorbidities (i.e., high blood pressure, diabetes, and obesity) were associated with a lower risk of discontinuation of the biologic DMARDs in patients with PsA (Alonso et al.). These findings may suggest that the effectiveness of this drug may be not influenced by the presence of comorbidity. In fact, the specific mechanism of action of secukinumab may not be influenced by the presence of associated disorder and possibly proposing its therapeutic relevance in a “real-life” setting. In this context, it must be pointed out that the evidence deriving from randomized clinical trials did not entirely clarify this issue. The strict enrolment criteria of these studies would decrease the generalizability of the results since the enrolled patients could not fully mirror the “real-life” scenario.

Taking together these findings, the increase of the rate of comorbidities, especially the cardiometabolic ones, is recognized in PsA with a consequent enhanced mortality and disability in these patients. In the clinical practice every rheumatologist routinely deals with these problems in patients with PsA and there are no specific therapeutic strategies to manage these comorbidities. This special issue has collected data about the influence of comorbidities, especially cardiometabolic, in the management of these patients. Thus, this special issue may provide the rationale of designing specific designed studies to fully evaluate these issues in PsA.

## Author Contributions

Both authors listed have made a substantial, direct, and intellectual contribution to the work and approved it for publication.

## Conflict of Interest

The authors declare that the research was conducted in the absence of any commercial or financial relationships that could be construed as a potential conflict of interest.

## Publisher's Note

All claims expressed in this article are solely those of the authors and do not necessarily represent those of their affiliated organizations, or those of the publisher, the editors and the reviewers. Any product that may be evaluated in this article, or claim that may be made by its manufacturer, is not guaranteed or endorsed by the publisher.
